# Melanoma detection, treatment, survival, and mortality through year 2 of the pandemic

**DOI:** 10.1007/s00403-024-03751-1

**Published:** 2025-01-09

**Authors:** Uriel Kim, Richard S Hoehn, Siran M Koroukian, Johnie Rose, Jeremy S Bordeaux, Bryan T Carroll

**Affiliations:** 1 School of Medicine, Case Western Reserve University, Cleveland, OH 44106 USA; 2 Department of Population and Quantitative Health Sciences School of Medicine, Case Western Reserve University, Cleveland, OH 44106 USA; 3 Case Comprehensive Cancer Center School of Medicine, Case Western Reserve University, Cleveland, OH 44106 USA; 4 Department of Internal Medicine, Cedars-Sinai Medical Center, Los Angeles, CA 90048 USA; 5 Division of Surgical Oncology, University Hospitals Cleveland Medical Center, Cleveland, OH 44106 USA; 6 Department of Dermatology, University Hospitals Cleveland Medical Center, Cleveland, OH 44106 USA; 7 Center for Community Health Integration School of Medicine, Case Western Reserve University, Cleveland, OH 44106 USA

**Keywords:** Melanoma, COVID-19, Incidence, Mortality, Survival

## Abstract

The COVID-19 pandemic affected the timely diagnosis and treatment of many cancers, including melanoma, the fifth most common cancer in the U.S. This study aimed to quantify the disruption and recovery of melanoma detection, treatment, survival, and mortality during the pandemic by analyzing data from the Surveillance, Epidemiology, and End Results (SEER) program from 2000 to 2021. Our epidemiological analysis found that melanoma incidence initially dropped by 14.8% (95% CI: − 17.2 to − 12.4) in 2020 compared to pre-pandemic projections. Although incidence rates substantially recovered by 2021, an estimated 10,274 patients (95% CI: − 12,824 to − 7,724) remained undiagnosed due to pandemic-related disruptions. Time-to-treatment and 1-year survival were mostly consistent with pre-pandemic trends, while melanoma-specific mortality modestly declined by 4.5% (95% CI: − 14.6 to 5.6) in 2021, though this was statistically non-significant. These findings suggest that healthcare systems adapted to the challenges posed by the pandemic, maintaining essential cancer services. However, the significant drop in melanoma diagnoses likely contributed to the observed reduction in mortality. Thus, re-establishing care for patients missed during the pandemic will be crucial to preventing a future increase in advanced-stage melanoma and related deaths.

**Supplementary Information:**

The online version contains supplementary material available at 10.1007/s00403-024-03751-1.

## Introduction

For patients with melanoma, successful management involves prompt detection and treatment, which were both likely adversely impacted by the COVID-19 pandemic. For example, analyses of nationally-representative cancer registry data, such as those from the Surveillance, Epidemiology, and End Results (SEER) Program, revealed that melanoma incidence fell by 15% during the first year (2020) of the pandemic, which was one of the largest drops among common cancers [1]. 

As disruptive as the COVID-19 pandemic was to the delivery of care, healthcare systems and patients also displayed a remarkable ability to adapt to the challenges and constraints posed by the pandemic [2]. Thus, the impact of the COVID-19 pandemic on melanoma care was likely highly dynamic [3]. Large-scale cancer registry data take several years to mature, as sufficient time must elapse to allow for the complete reporting of cancer cases as well as for the adequate accrual of follow-up time [4, 5]. Thus, it is only now possible to utilize these large-scale cancer registry data to evaluate the impact of COVID-19 on melanoma care beyond the first year of the pandemic.

In this study, we sought to evaluate the dynamic impact the COVID-19 pandemic has likely had on melanoma care by conducting an epidemiological analysis of the latest April 2024 release of SEER data, which includes data through the first two years (2020–2021) of the pandemic. To gain a comprehensive understanding of the impact of the pandemic on melanoma outcomes, we analyzed four important quantitative, epidemiological measures of melanoma burden: incidence per 100,000, time to first definitive treatment, short-term (1-year) survival, and stage-specific mortality. Since melanoma is the fifth most common cancer in the US [6], understanding the magnitude of the disruption that the pandemic caused on melanoma outcomes – and the degree to which they recovered – can lend important insight into the overall impact that the pandemic has had on cancer-related morbidity and mortality.

## Materials and methods

We first quantified the disruption and recovery of melanoma detection (incidence) associated with the COVID-19 pandemic. Utilizing a method described more extensively elsewhere [1], we compared the expected versus observed incidence during the first (2020) and second years (2021) of the pandemic. The expected incidence in 2020 and 2021 were estimated by modeling trends in incidence from 2000 to 2019 using joinpoint regression [7–9] and then using those trend models to project forward the expected incidence for 2020 and 2021. The percent difference between the expected and observed melanoma incidence in 2020 and 2021 captured the “disruption” and the corresponding “recovery” in melanoma detection, respectively. These figures were estimated for select demographic subgroups (sex, race/ethnicity, age group) and cancer stage. To further contextualize the findings, we estimated the deficit in detected cases at the national level. These estimates were modelled using the percent difference figures for 2020 and 2021 as well as the case count data from SEER, extrapolated to the national level (SEER covers 48% of the US population [10]). The data were accessed via SEER*Stat software [11], with incidence rates being age-adjusted and delay adjusted [4] when appropriate.

A similar approach was utilized to evaluate the impact of the pandemic on timely treatment of melanoma. We developed a trend model trained on ten years (2010–2019) of time-to-treatment (TTT) data using joinpoint regression before the pandemic to project forward the expected mean TTT in each of the first two years of the pandemic. These expected means were compared to the observed means in 2020 and 2021. We conducted subgroup analyses by stage and race / ethnicity, controlling all our estimates for year-to-year variations in age, sex, and when applicable, for stage and race / ethnicity. TTT from SEER was pre-calculated, since the underlying month and day of diagnosis and treatment data fields are not routinely available to researchers for analysis (only year of diagnosis was available). Thus, for a more granular perspective, we additionally conducted a month-by-month analysis of TTT using a state cancer registry (Ohio), since the month and day of diagnosis and treatment were available to us under a data use agreement.

Our survival analyses focused on short-term survival, given that sufficient follow-up time for long-term survival (i.e. 5-years) has not yet accrued for melanomas diagnosed in 2020 and 2021. The longest survival interval available for analysis in SEER for patients diagnosed with melanoma in 2020 and 2021 was 11-month survival, which was assumed to approximate 1-year survival. Given that more localized melanomas have a relatively indolent course for which short-term (1-year) survival statistics have relatively limited clinical utility, we focused the survival analyses on metastatic melanomas only. We once again utilized the same approach for these survival analyses as we did for the incidence and TTT analyses, comparing the expected versus observed 1-year survival in 2020 and 2021. Due to the statistical rarity of metastatic melanoma among certain groups, subgroup analyses were only possible by sex and not by race / ethnicity.

Finally, we conducted mortality analyses (melanoma-specific deaths per 100,000). To clarify the specific impact that the pandemic had on mortality, we again focused our analyses on patients diagnosed with metastatic melanoma, and further limited our sample to those who died within one year of diagnosis. Restricting the sample to this population ensured that the observed melanoma-specific deaths represented patients who were diagnosed and received treatment during the pandemic. We isolated melanoma-specific deaths occurring in patients diagnosed with metastatic melanoma using an approach called incidence-based-mortality (IBM), developed by the National Cancer Institute [12]. Traditional cancer-specific mortality statistics are derived from death certificates only, which lack important clinical information (i.e. stage-at-diagnosis) of the decedent. In contrast, in IBM, death certificates are linked with cancer registry data, and thus contain additional clinical information about the incident tumor that led to death. The IBM approach is described more extensively elsewhere [12, 13]. As previously, a joinpoint trend model was fitted to the data (this time, trained on mortality data from 2005 to 2020) to project forward the expected mortality rate in 2021. Due to statistical (power) limitations, no subgroup analyses were performed.

## Results

The analyses of incidence included *N* = 673,681 patients diagnosed with melanoma between the years 2000–2021.

**Fig. 1:**
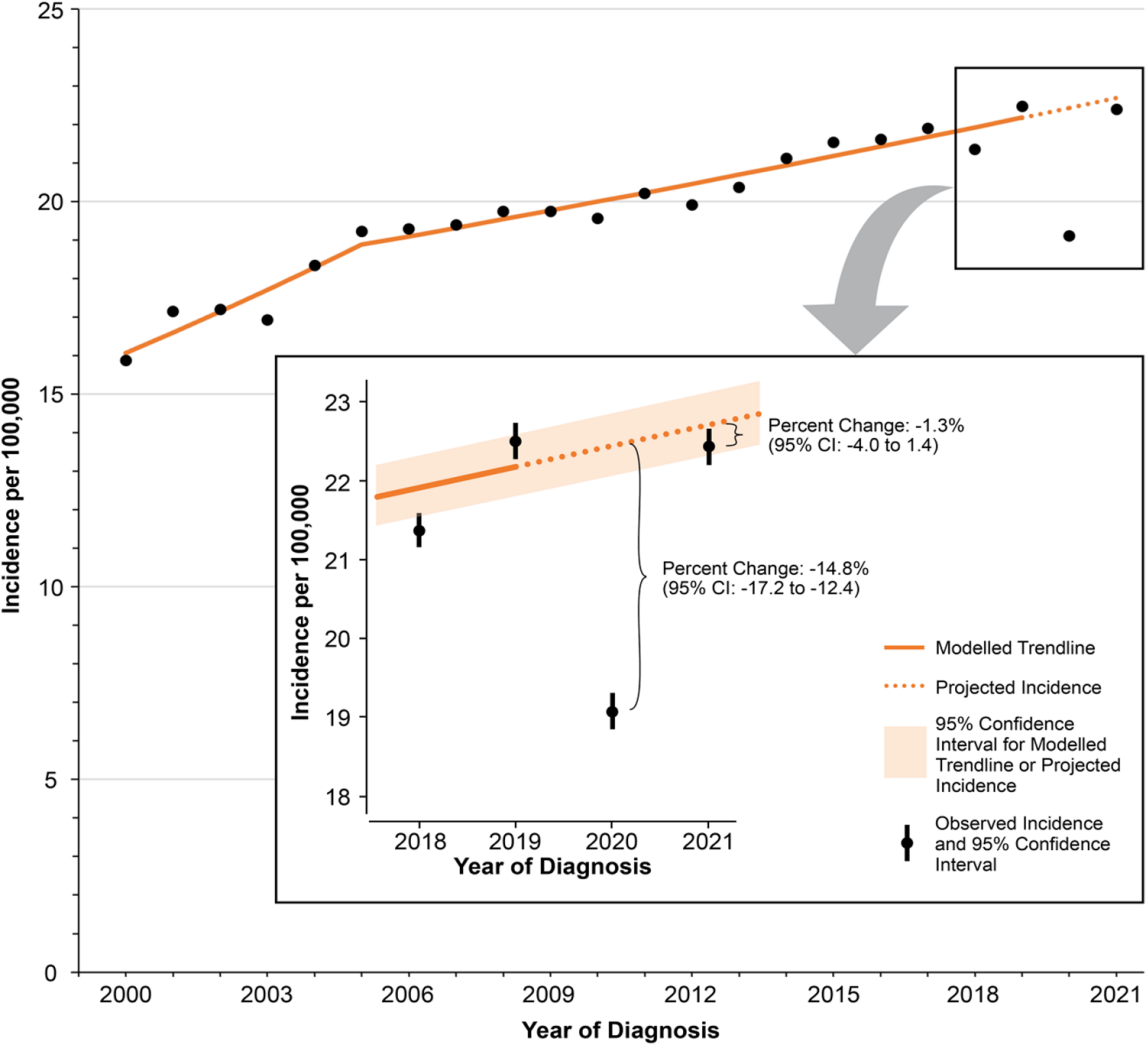
Disruption and recovery of melanoma incidence during the COVID-19 pandemic

The joinpoint trend analysis revealed that there have been two distinct trends in incidence during the study period (Fig. 1): first, between 2000 and 2005, melanoma incidence has been increasing by 3.27% per year (*p *< 0.001), while between 2005 and 2019, melanoma incidence has been increasing, albeit at a slower rate, of 1.16% per year (*p *< 0.001). Given that the modelled incidence of melanoma in 2019 was 22.17 per 100,000, applying the 1.16% annual growth rate in melanoma would have translated to an expected incidence of 22.43 and 22.69 per 100,000 in 2020 and 2021, respectively. However, the actual observed incidence rate was 19.11 and 22.40 per 100,000 and in 2020 and 2021, respectively. Thus the percent difference between the expected and observed incidence in 2020 and 2021 was − 14.8% (95% CI: − 17.2 to − 12.4) and − 1.3% (95% CI: − 4.0 to 1.4), respectively (Table 1)

**Table 1:** Stratified analysis of the disruption and recovery of melanoma incidence during year 1 and 2 of the pandemic

	Pandemic year 1 (2020)				Pandemic year 2 (2021)				Cumulative (year 1 & 2) deficit/surplus of expected cases, (95% CI)
	Observed incidence per 100,000	Observed cases (US estimate)	Percent difference from expected incidence	Deficit/surplus of cases from expected cases	Observed incidence per 100,000	Observed cases (US estimate)	Percent difference from expected incidence	Deficit/surplus of cases from expected cases	
All Invasive	19.11	**71**,**441**	**− 14.8%**	**− 9**,**210**	22.40	83,685	− 1.3%	− 1,064	**− 10**,**274** (− 12,824 to − 7,724)
Localized	14.26	54,088	**− 17.3%**	**− 7**,**208**	17.07	65,598	− 1.9%	− 833	**− 8**,**041** (− 9,369 to − 6,713)
Regional	1.89	7,106	**− 9.1%**	**− 536**	2.06	7,910	− 2.0%	− 106	**− 643** (− 1,000 to − 285)
Distant	0.90	3,471	**− 10.0%**	**− 287**	0.95	3,706	**− 1.8%**	**− 43**	**− 330** (− 354 to − 306)
Sex									
Female	15.44	29,685	**− 14.0%**	**− 3**,**551**	18.19	34,884	− 0.2%	− 39	**− 3**,**591** (− 4,718 to − 2,463)
Male	24.25	41,755	**− 16.2%**	**− 5**,**658**	28.33	48,801	**− 3.2%**	**− 1**,**025**	**− 6**,**683** (− 7,866 to − 5,501)
Race / Ethnicity									
Hispanic (any race)	4.06	1,693	**− 12.1%**	**− 171**	4.76	2,052	2.4%	20	**− 151** (− 243 to − 59)
NH AI / AN	8.35	194	− 20.1%	− 30	11.56	244	6.5%	7	**− 23** (− 84 to 37)
NH Asian / PI	1.08	225	**− 17.3%**	**− 31**	1.30	276	0.0%	0	**− 31** (− 54 to − 8)
NH Black	0.92	337	− 6.9%	− 20	0.99	368	0.0%	0	− 20 (− 66 to 25)
NH White	28.24	68,992	**− 16.2%**	**− 8**,**958**	33.07	80,745	− 3.6%	− 1,091	**− 10**,**049** (− 11,940 to − 8,158)
Age Group									
< 20	0.23	182	**− 21.1%**	**− 31**	0.24	186	**− 13.2%**	**− 14**	**− 44** (− 63 to − 26)
20–39	5.92	4,819	**− 13.2%**	**− 543**	6.71	5,414	− 0.1%	− 2	**− 546** (− 629 to − 462)
40–64	23.33	25,677	**− 14.0%**	**− 3**,**055**	27.07	29,224	− 0.7%	− 132	**− 3**,**187** (− 3,788 to − 2,585)
65+	81.34	40,764	**− 16.5%**	**− 5**,**581**	99.60	48,861	− 3.1%	− 916	**− 6**,**497** (− 8,283 to − 4,711)

*NH *non-hispanic, *AI/AN* American Indian/Alaska native, *PI *Pacific Islander

Bolded figures indicate statistically significant (*p* < 0.05) values

When extrapolating these percent difference estimates to the national population, the estimated cumulative (2020–2021) deficit in diagnosed cases was 10,274 (95% CI: − 12,824 to − 7724). Analyses by stage revealed that earlier-stage (localized, regional disease) melanoma incidence decreased substantially in 2020 but recovered within pre-pandemic projections by 2021. Distant-stage (metastatic) melanoma incidence also fell in 2020, with detection remaining significantly, albeit mildly, depressed into 2021. The demographic subgroup analyses revealed that disruptions were primarily concentrated during the first year of the pandemic. By 2021, most incidence rates for demographic subgroups were consistent with pre-pandemic projections. Notable exceptions were male patients (percent difference: − 3.2%, 95% CI: − 6.1 to − 0.3) and younger (< 20 years) patients (percent difference: − 13.2%, 95% CI: − 26.0 to − 0.4).

**Fig. 2:**
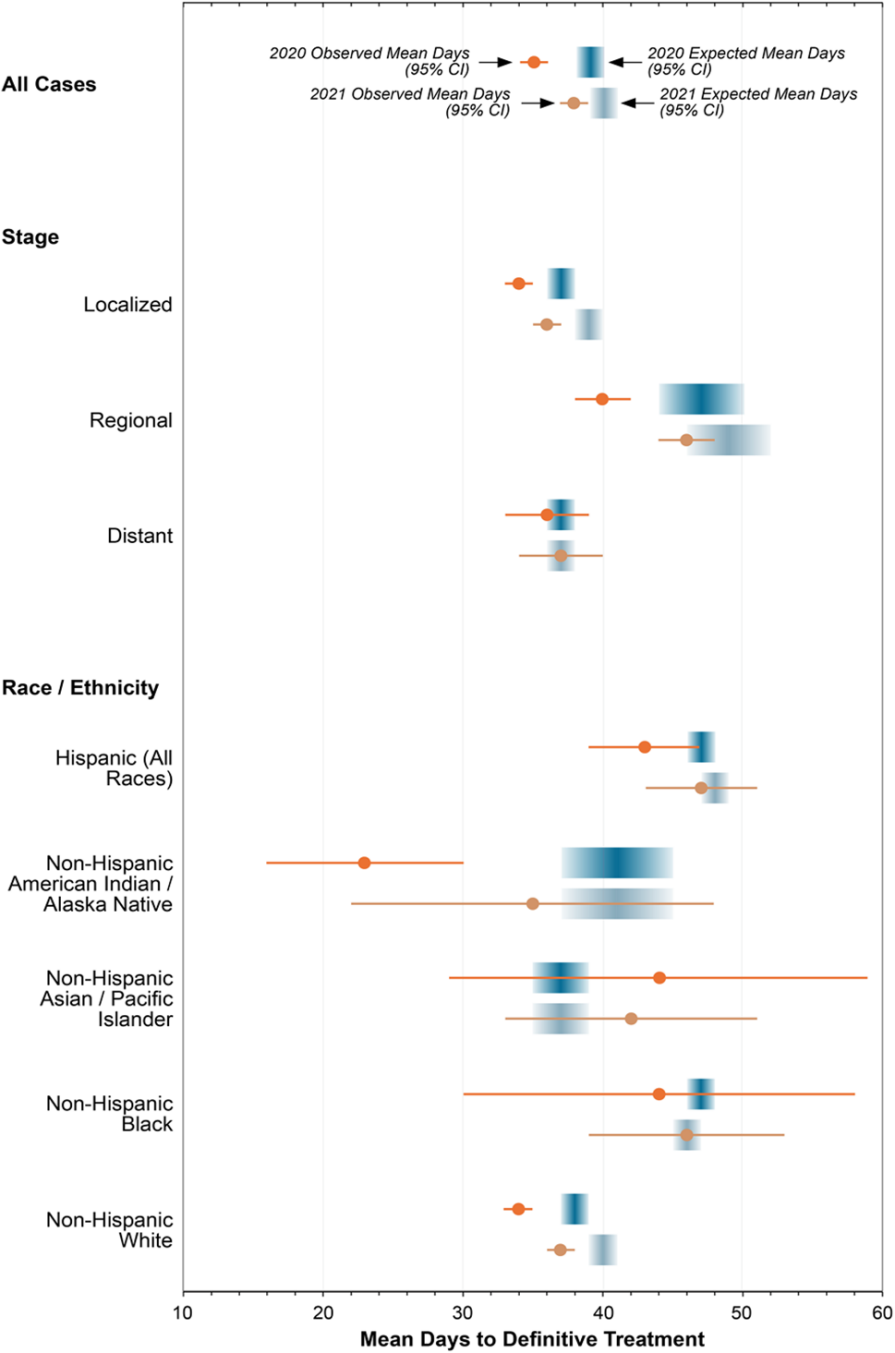
Observed and expected mean time to treatment during year 1 (2020) and year 2 (2021) of the pandemic

The analyses of TTT included *N* = 110,098 patients treated for melanoma between the years 2010–2021. Joinpoint analyses revealed that TTT had most recently been increasing at an annual rate of 3.47% (*p* < 0.001), meaning that the expected mean time to first definitive treatment was 39 (95% CI: 38–40) and 40 days (95% CI: 39–41) days during Year 1 and Year 2 of the pandemic respectively. However, the observed mean TTT was statistically shorter at 35 (95% CI: 34–36) and 38 days (95% CI: 37–39), respectively. The stage-specific and race / ethnicity subgroup analyses generally revealed similar findings of non-clinically meaningful changes (Fig. 2). An exception was non-Hispanic American Indian / Alaska Native patients, whose mean TTT decreased by 18 days in 2020 (*N* = 14), though this finding should be interpreted cautiously given the small sample size. The month-by-month analysis of state-level data (*N* = 4,824 for patients treated between 2018 and 2021) also failed to detect a clear pattern of clinically meaningful differences in TTT (Supplemental Fig. 1), including during the months most impacted by the pandemic “lockdowns” (March-April 2020).

The analyses of 1-year survival included *N* = 16,647 patients diagnosed with metastatic melanoma between the years 2004–2021. During Year 1 and Year 2 of the pandemic, the expected 1-year survival was 53.16% (95% CI: 56.11–53.18%) and 52.19% (95% CI: 55.09–52.21%) respectively (Table 2). The observed 1-year survival was similar in 2020 to the expected survival, but greater in 2021, increasing by 3.90% points on an absolute basis to 56.09%, or a relative 7.5% improvement (95% CI: 2.2–12.7%). The sex-stratified analysis suggests that many of the improvements in survival in 2021 were driven by female patients, who saw their observed survival increase by 20.1% from expected (95% CI: 13.7–26.6).

**Table 2.**
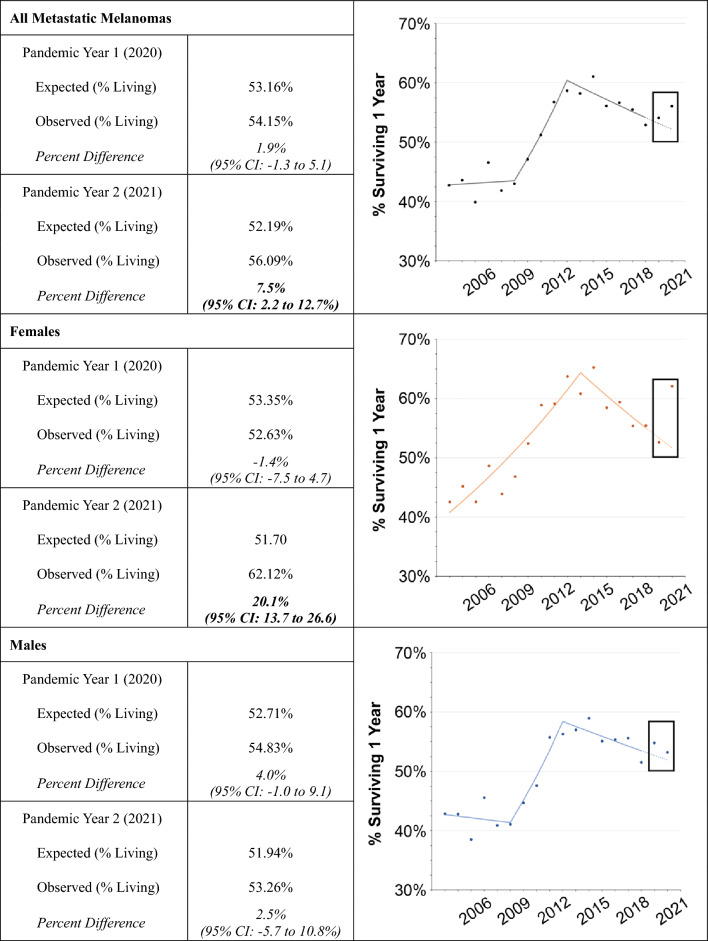
Percent of patients with metastatic melanoma surviving 1 year during the pandemic

**Fig. 3:**
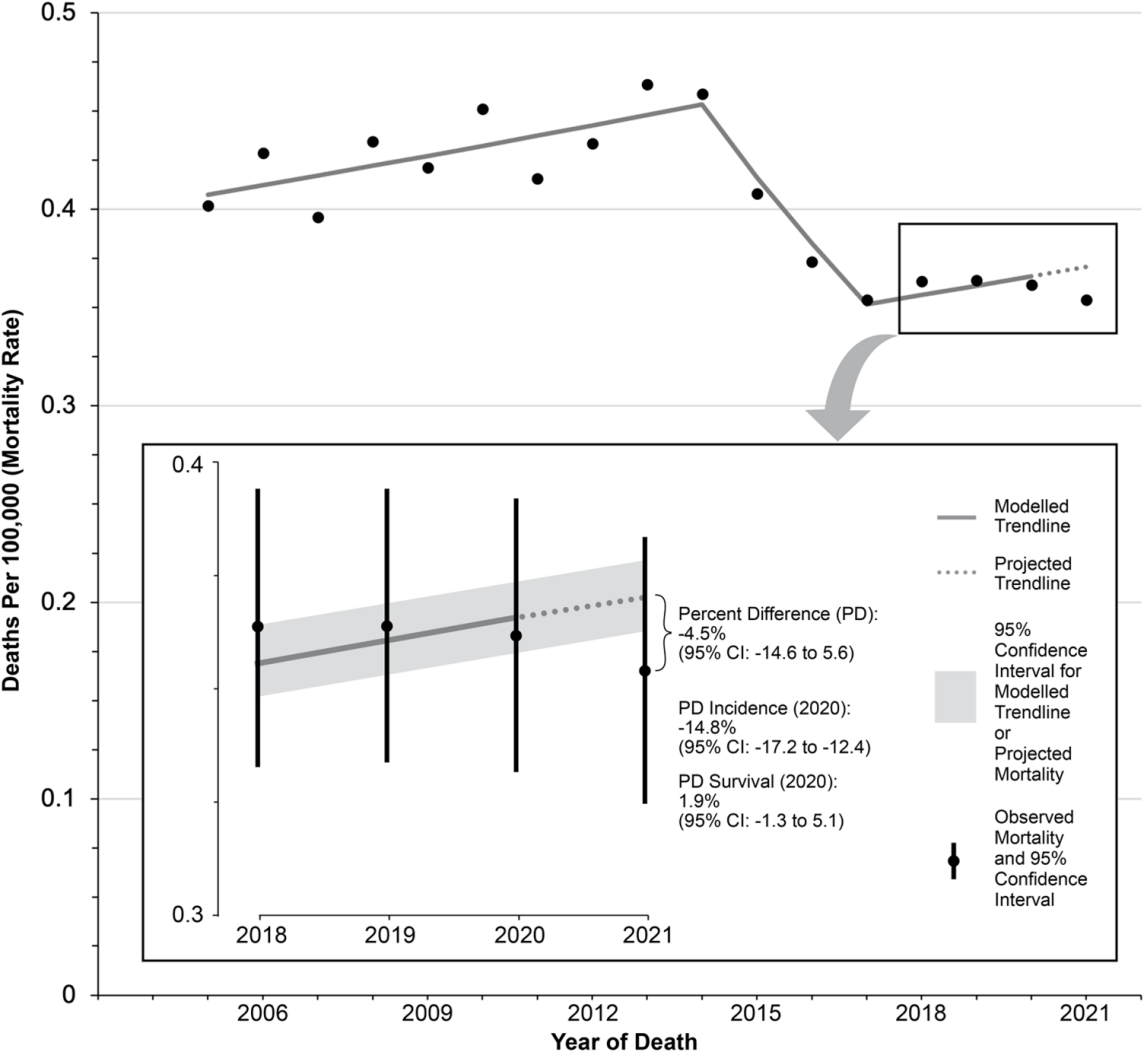
Observed and expected mortality among patients with metastatic melanoma who die within year of diagnosis

Finally, the melanoma-specific mortality analysis of patients diagnosed with metastatic melanoma who died within one year of diagnosis (and died between 2005 and 2021) included *N* = 9,509 patients. In 2021, there were 0.35 per 100,000 observed deaths, representing people who were diagnosed with metastatic melanoma during the pandemic (2020, 2021) and died within a year. This compared to 0.37 per 100,000 expected deaths, representing a relative reduction (albeit non-significant) of 4.5% (95% CI: −14.6 to 5.6) in the mortality rate (Fig. 3).

## Discussion and conclusion

Incidence, time-to-treatment, survival, and mortality are key epidemiological measures that allow for comprehensive surveillance of cancer burden. Incidence captures the ability of patients to receive preventive care, screening services, and timely diagnostic care; time-to-treatment captures challenges related to patients receiving specialized cancer services; and survival captures the efficacy of treatments. All these epidemiological measures predicate mortality, which is the ultimate measure of cancer burden on a population level, capturing the number of deaths attributable to a particular cancer [14, 15]. The COVID-19 pandemic likely impacted all these measures, and this analysis provides the first epidemiological analysis of nationally representative cancer registry data for the specific impact that COVID-19 had on melanoma during the first (2020) and second (2021) years of the pandemic.

The analyses of melanoma incidence revealed that while melanoma incidence rates recovered to pre-pandemic projections in 2021, detection rates need to improve further to address the substantial number of patients who were “missed” during the early part of the pandemic. Indeed, by the end of 2021, an estimated 10,274 patients (95% CI: − 12,824 to − 7724) went undiagnosed with melanoma because of the pandemic. Nonetheless, this interval recovery reflects a remarkable degree of adaptability and resilience on the part of healthcare systems and patients. From the perspective of healthcare systems, the robust recovery of melanoma incidence rates could reflect dermatology appointment allocation processes which were developed during the pandemic that prioritized patients requiring diagnostic care for possible malignancies [16, 17]. 

The analyses of treatment delayed revealed that the pandemic did not appear to have a clinically meaningful impact on time-to-treatment in the first two years of the pandemic. Additionally, the pandemic did not appear to exacerbate existing disparities in time-to-treatment by race and ethnicity [18]. Minimal differences between stage groups also suggest that patients were able to get needed treatment for melanoma, regardless of the modality of the primary treatment (wide excision for localized and regional disease or systemic therapy for regional and advance disease). The SEER dataset analyzed did not capture the specific modality patients received, though the literature seems to support that patients were able to continue to receive guideline concordant care for melanoma during the pandemic, especially for regional and advanced disease [19, 20]. Importantly, however, the ability of patients to receive timely care during the pandemic may have been at least partially facilitated by a decrease in patient volumes due to a dramatic drop in the detection and diagnosis of melanoma, especially in 2020.

A similar logic – a large reduction in patient volumes facilitating the delivery of needed cancer services despite the barriers posed by the pandemic – may explain the lack of adverse changes observed for 1-year survival in metastatic melanomas. In fact, in 2021, survival appeared to improve significantly compared to prevailing trends, particularly for women. We note, however, that 1-year relative survival in 2021 appeared to be consistent overall with step change improvements in survival that occurred during the late 2000’s and early 2010’s, coincident with the registrational trials (and ultimate approval) of paradigm-shifting targeted and immunologic therapies for metastatic melanoma such as vemurafenib and ipilimumab [21, 22]. The degree to which survival truly improved during the pandemic is more difficult to ascertain compared to incidence given the larger year-to-year variability in survival, which make trend modeling and estimating projected survival in 2020 and 2021 more challenging.

Finally, for the mortality analysis, we observed a non-significant reduction in mortality rate in 2021 of 4.5% (95% CI: − 14.6–5.6). These mortality statistics specifically apply to individuals diagnosed with metastatic melanoma with a survival time of 12 months or less, so the deaths from melanoma observed in 2021 were from patients who were diagnosed during 2020 and 2021. Focusing on patients with less than a year of survival was necessary to isolate the specific impact of the pandemic on mortality. Without any limitations on survival time, mortality rates observed during the pandemic would otherwise include a substantial number of patients who were diagnosed and treated for their melanoma many years before the onset of the pandemic. Thus, inclusion of these patients would bias the result towards the null. In the setting of metastatic melanoma, including only deaths that occur within one year of diagnosis captures a majority of melanoma-specific deaths, given the aggressive course of metastatic melanoma even with treatment. The aggressive nature of metastatic melanoma is captured quantitatively in “burn-in” [13] analyses (Supplement Fig. 2) and conditional survival analyses [23] (Supplemental Fig. 3) which reveal that nearly all melanoma-specific deaths occur within three years of diagnosis, while the majority melanoma-specific deaths occur in the first year.

Declines in cancer-specific mortality is a function of changes in incidence (fewer diagnosed cases of melanoma will lead to fewer cases of melanoma-specific deaths) and survival (increased survival will reduce the number of melanoma-specific deaths) [12]. In this instance, the decline in mortality appears to be primarily driven by a decrease in incidence rather than improvements in survival during the pandemic, as the relative reduction in incidence (−14.8% in 2020) was far greater than the relative improvement in survival (1.9% in 2020). Thus, in the near term, melanoma mortality statistics are likely to continue to remain stable or even fall. However, this ostensibly positive trend in melanoma burden should be interpreted cautiously since it primarily is driven by individuals who could not receive diagnostic care during the pandemic. Thus, the near-term drop or stability in melanoma-specific mortality would predict a surge of melanoma-specific deaths in the long-term, as patients who were missed during the pandemic present in future years with more advanced, aggressive disease. Thus, re-establishing care for the estimated 10,274 patients missed during the pandemic will be essential to reduce the long-term impact of the COVID-19 pandemic on melanoma-specific mortality. Other studies examining changes in melanoma detection at the community-level have found that communities with a greater degree of socioeconomic disadvantage experienced larger disruptions in melanoma detection [24], so re-establishing care will also be crucial to prevent the widening of disparities.

Though the global scale of the COVID-19 pandemic was unprecedented, previous regionally cataclysmic events such as natural disasters and geopolitical conflicts that disrupted routine care for cancer are potentially illustrative. During these events, important facets of re-establishing care for potentially missed patients included clear communication to the public about the magnitude of the disruption in cancer care, adequate provisioning of healthcare resources to deal with a potential surge in demand for cancer services, as well as the targeted deployment of resources to the communities and subpopulations most heavily impacted [25–27]. 

The key limitation of this study is that sufficient follow up time has yet to elapse for some of the pandemic-associated epidemiologic data to mature. Incidence is the fastest data to mature, so incidence rates for the years 2020 and 2021 faithfully reflect health systems changes that occurred during the pandemic which may have impacted cancer detection and diagnosis. In contrast, other statistics, such as survival and mortality, take longer to mature. For example, 5-year survival, the traditional measure of cancer survivorship, will not be available for 2020 and 2021 for three to four more years since adequate follow-up time has not yet accrued. We surmounted the issue of limited follow-up time by focusing on metastatic melanoma, the most aggressive form of melanoma, and limited our survival and mortality analyses to those with 1-year of survival time. While this relatively short follow-up time provides a reasonably complete and clinically relevant understanding of the impact of the pandemic in metastatic melanoma, it would not be appropriate for other early-stage melanomas with a more indolent course. Thus, continued epidemiologic surveillance is necessary to better understand the impact of the pandemic on survival and mortality in these melanomas.

In conclusion, this epidemiological analysis of nationally representative cancer registry data revealed that while COVID-19 was initially disruptive to the detection and diagnosis of melanoma, incidence largely recovered to pre-pandemic projections by the second year (2021) of the pandemic. Additionally, the pandemic appeared to have a limited impact on time-to-treatment and 1-year survival, reflecting the resilience of the health care system and patients to deliver and receive needed cancer care. However, the delivery of these limited and constrained healthcare services during the pandemic was likely facilitated by a large drop in the volume of melanoma patients, with an estimated 10,274 patients going undiagnosed because of the pandemic. Re-establishing care for these missed patients is essential to averting a surge of melanoma-specific deaths in the long-term, as these patients may present in the future with more advanced melanoma As updated data become available, especially long-term follow-up data, trends in incidence, survival, and mortality should be followed to gain a comprehensive understanding of the impact of COVID-19 on the burden of melanoma.

## Supplementary Information

Below is the link to the electronic supplementary material.

Supplementary material 1 (DOCX 500.0 kb)

## Data Availability

Data is available upon request, with access moderated by the Surveillance, Epidemiology, and End Results Program.
