# Supplementary material for: Melanoma detection, treatment, survival, and mortality through year 2 of the pandemic
Source: Arch Dermatol Res. 2025 Jan 9;317(1):209. doi: 10.1007/s00403-024-03751-1 (PMC11717807; doi:10.1007/s00403-024-03751-1)
Supplement: Supplementary file 1 — Supplementary material 1 (DOCX 500.0 kb) [file 403_2024_3751_MOESM1_ESM.docx]

**Supplemental Figure 1.** State-level (Ohio) Analysis of Month-by-Month Time to Treatment and Melanoma Case Volumes


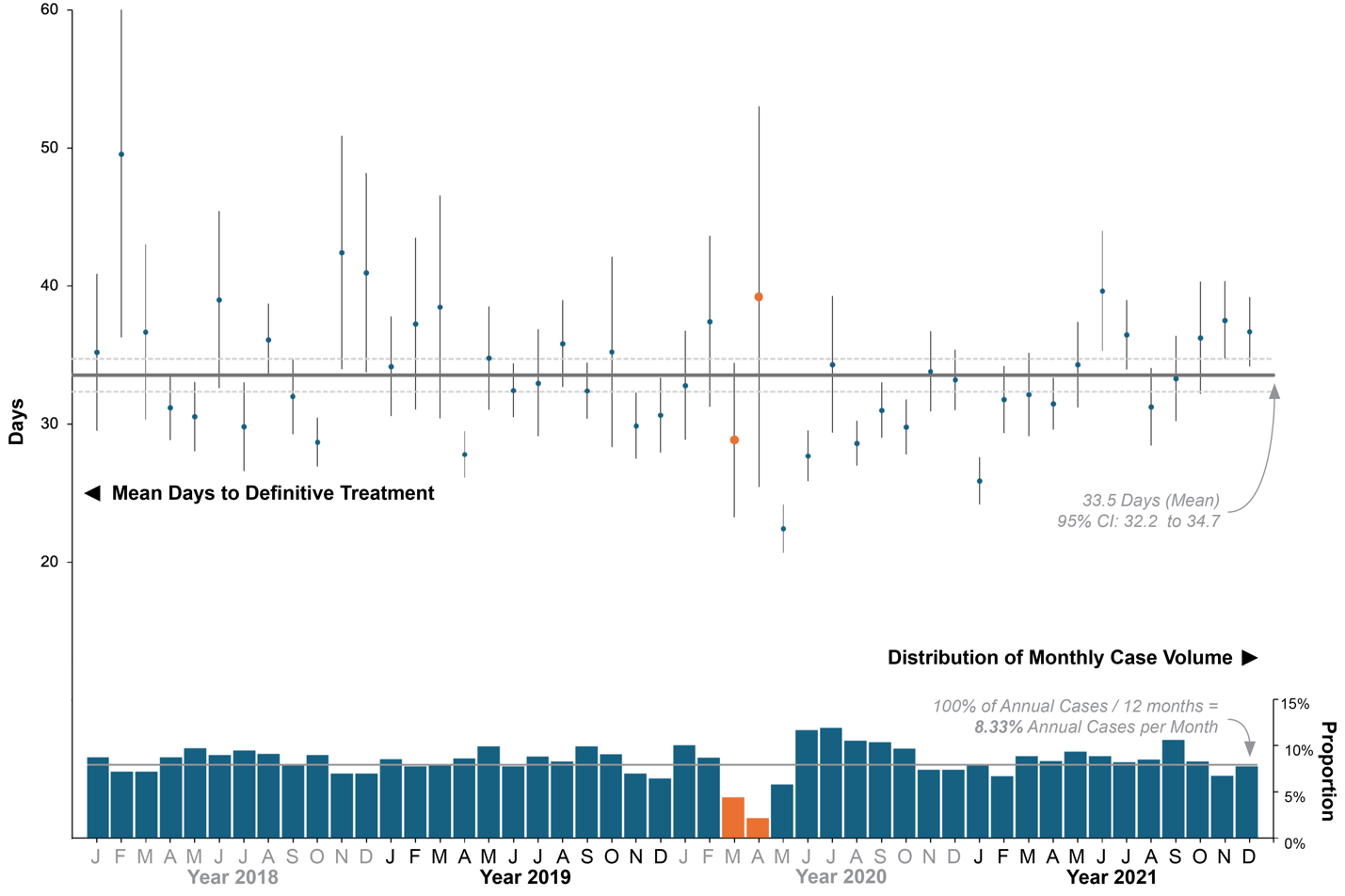


The above figure represents the month-by-month analysis of TTT using a state cancer registry (Ohio). Such an analysis was not possible using SEER since month of diagnosis is not routinely available to researchers. This analysis included N= 4,824 patients, who were diagnosed with melanoma in Ohio between the years 2018-2021. This analysis failed to detect a clear pattern of clinically meaningful differences in TTT, including during the months most impacted by the pandemic “lockdowns” (March-April 2020, indicated in the orange dots). The ability of patients to receive timely care during the pandemic was at least partially facilitated by a decrease in patient volumes due to a dramatic drop in the detection and diagnosis of melanoma during the pandemic lockdowns (indicated in the orange bars).

**Supplemental Figure 2.** Mortality Estimates for Melanoma Based on Data from Death Certificates versus Incident Metastatic Melanoma Cases to Determine “Burn-in” Period


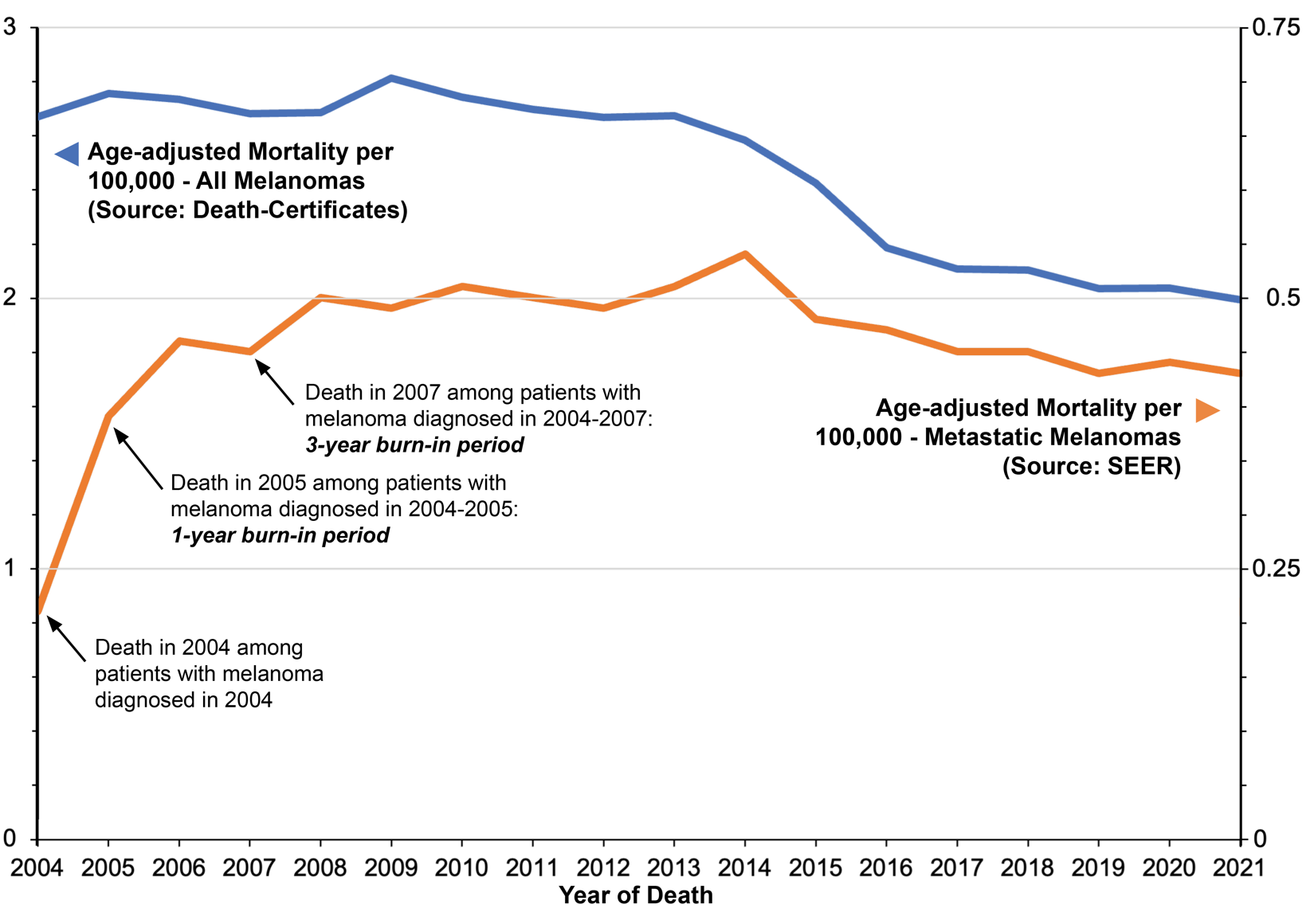


The blue line indicates melanoma-specific mortality per 100,000 estimated from death certificates nationally (data compiled by the Centers for Disease Control and Prevention’s National Center for Health Statistics), while the orange line indicates melanoma-specific mortality for melanomas diagnosed at the metastatic stage, as estimated using the incidence-based mortality method using SEER. Note that the two lines are plotted on two different scales. This exercise helps determine the “burn-in” period for a specific kind of cancer (ie. metastatic melanoma), or the years required to capture the majority of deaths attributable to a cancer diagnosed in a given year. The burn-in period is determined by examining when the mortality rate estimated from the incidence-based mortality method appears to plateau and parallel the mortality rate estimated from the National Center for Health Statistics. In this instance, it appears that that optimal burn-in period is around 3 years, though a shorter burn in period of around 1 year captures a majority of metastatic-melanoma related deaths.

**Supplemental Figure 3.** Relative Survival Rates by Time Since Diagnosis to Validate 1-Year Burn-in Period


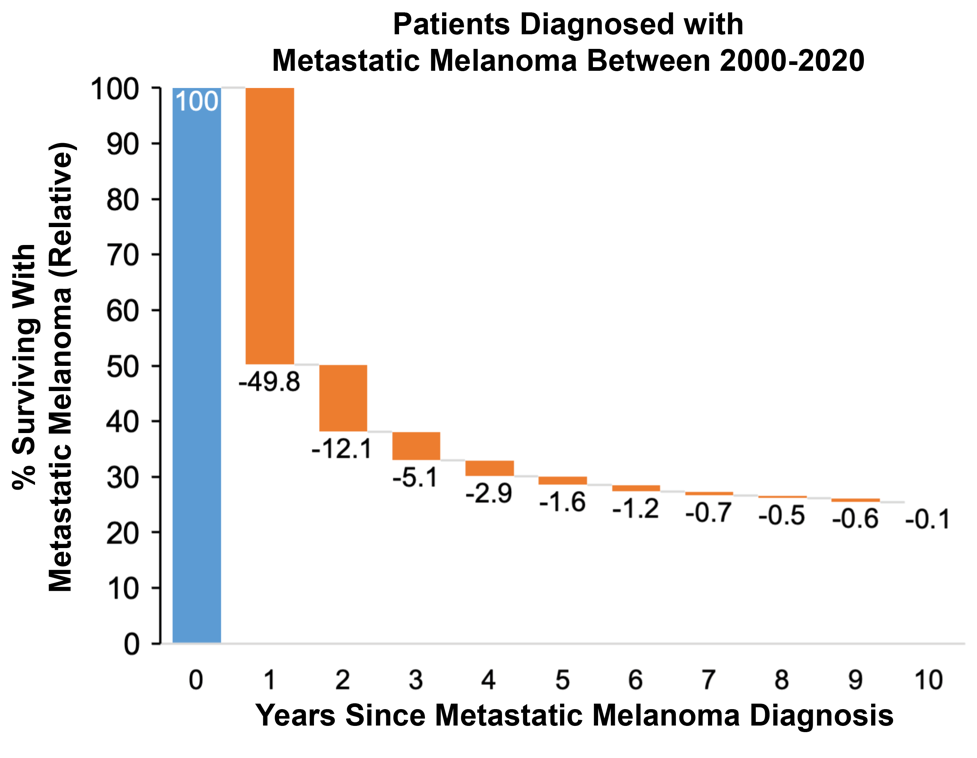


The above figure indicates the relative survival rates by time (in years) since diagnosis, estimated from the SEER data for metastatic melanomas diagnosed between 2000-2020. Immediately at diagnosis, survival is 100%, after one year of diagnosis, only 50.2% of patients are alive (on a relative basis) among those diagnosed with metastatic melanoma. After 2 years since diagnosis, only 38.1% of patients are alive, while after 3-year since diagnosis, only 33.0% of patients are alive. After 3 years since diagnosis, deaths attributable to a patient’s melanoma plateau. This validates a 3-year burn-in period as being appropriate to capture nearly all deaths attributable to metastatic melanomas, while a burn in period of 1-year captures the majority of deaths attributable to metastatic melanomas.
